# Association of Matrix Metalloproteinase-2 mRNA Expression with Subtypes of Pediatric Cholesteatoma

**DOI:** 10.1155/2021/6644897

**Published:** 2021-03-10

**Authors:** Taichi Kan, Hiromi Ueda, Taishi Takahara, Yoshimasa Tsuchiya, Mayuko Kishimoto, Yasue Uchida, Tetsuya Ogawa, Wataru Ohashi, Toyonori Tsuzuki, Yasushi Fujimoto

**Affiliations:** ^1^Department of Otorhinolaryngology, Aichi Medical University School of Medicine, 1-1, Yazakokarimata, Nagakute, Aichi 480-1195, Japan; ^2^Department of Otorhinolaryngology, Nagoya Ekisaikai Hospital, 4-66, Shonencho, 11 Nakagawa-ku, Nagoya, Aichi 454-0854, Japan; ^3^Ear Surgical Center, Meitetsu Hospital, 2-26-11, Sako, Nishi-ku, Nagoya, Aichi 451-8511, Japan; ^4^Department of Surgical Pathology, Aichi Medical University Hospital, 1-1, Yazakokarimata, Nagakute, Aichi 480-1195, Japan; ^5^Division of Biostatistics, Clinical Research Center, Aichi Medical University School of Medicine, 1-1, Yazakokarimata, Nagakute, Aichi 480-1195, Japan

## Abstract

**Objective:**

Cholesteatoma is a clinically heterogeneous disease, with some patients showing spontaneous regression, while others experiencing an aggressive, lethal disease. Cholesteatoma in children can be divided into two types: congenital and acquired. Identifying good prognostic markers is needed to help select patients who will require immediate surgical intervention. Matrix metalloproteinase-2 (MMP2) was previously reported to play an important role in cholesteatoma progression, by promoting bone destruction and keratinocyte infiltration. Herein, we analyzed *MMP2* mRNA expression level in cholesteatoma using RNA-in situ hybridization in formalin-fixed, paraffin-embedded (FFPE) tissue samples.

**Methods:**

Sixty patients with cholesteatoma under 15 years old, who underwent their primary surgery at Aichi Medical University's Otolaryngology Department, were analyzed for MMP2 expression level, using RNA-in situ hybridization.

**Results:**

There were no significant differences in *MMP2* mRNA expression level between congenital cholesteatoma and acquired cholesteatomas. In congenital cholesteatoma, higher MMP2 signals were observed in the open type than in the closed type (*p* < 0.001). In acquired cholesteatoma, higher MMP2 signals were observed in the pars tensa than in the pars flaccida (*p* < 0.001). *MMP2* mRNA expression level was almost exclusively found in the fibroblasts or in the inflammatory cells in the stroma, but not in the epithelium.

**Conclusion:**

Our study reveals that *MMP2* mRNA expression level is strongly associated with the subtypes of cholesteatoma. The findings suggest that the level of expression of *MMP2* mRNA may be related to the pathogenesis and aggressive features of cholesteatoma.

## 1. Introduction

Cholesteatoma is defined as a nonneoplastic lesion, characterized by the accumulation of keratin produced by the stratified squamous epithelium in the middle ear accompanied by chronic inflammation. Cholesteatoma in children can be divided into two types: congenital and acquired cholesteatoma. Congenital cholesteatoma is further classified into the closed type, characterized by epithelial cysts without keratin exposure, and the open type, characterized by ruptured cyst and proliferation of flat keratinizing epithelium. Acquired cholesteatoma is classified into the pars tensa type and pars flaccida type based on its location. Acquired cholesteatoma is thought to be caused by the retraction of the eardrum, while congenital cholesteatoma is hypothesized to derive from an ectopic epidermoid at rest or ingrowth of meatal epidermis [1–4]. Although most cholesteatomas are a benign and slowly progressing disease requiring surgical resection, a minor population of congenital cholesteatoma cases spontaneously regresses several months after diagnosis; therefore, it is difficult to predict the clinical behavior of cholesteatoma [5]. Consequently, determining an appropriate follow-up period duration for patients with cholesteatoma is critical, and good prognostic markers are needed to aid in selecting patients who will require immediate surgical intervention.

Several studies have suggested that certain cytokines are associated with the pathogenesis or progression of cholesteatoma [1, 6–8]. Among them, matrix metalloproteinase-2 (MMP2) was reported to play an important role in cholesteatoma progression, by promoting bone destruction and keratinocyte invasion [7, 9, 10]. However, previous studies have focused on quantifying serum MMP2 [10], and MMP2 expression level in cholesteatoma lesions has not been well investigated.

In this study, we evaluated clinical samples to analyze the association of *MMP2* mRNA expression level with different subtypes of cholesteatoma.

## 2. Materials and Methods

### 2.1. Study Design and Measurements

From July 2009 to May 2017, 98 patients under 15 years of age with cholesteatoma, who underwent the primary surgery at Aichi Medical University's Otolaryngology Department, were included in this study. After excluding 38 cases due to microspecimens, samples without epithelial components, secondary properties, or unclassifiable specimens, 60 cases were analyzed in this study. None of the patients had received any treatment before surgery. Residual rate was defined as the ratio of patients who were found to have residual lesions or recurrence after primary surgery.

**Figure 1:**
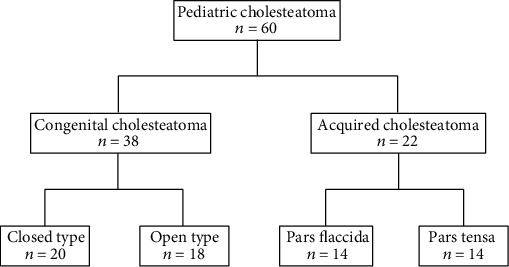
Flowchart of patient selection.

The classification of congenital cholesteatoma and acquired cholesteatoma was based on the Japan Otological Society (JOS) classification [11]: there were 38 congenital cholesteatoma cases (closed type: 20 cases and open type: 18 cases) and 22 acquired cholesteatoma cases (pars flaccida type: 14 cases and pars tensa type: 8 cases) (Figure 1). Eight normal skin samples from pediatric accessory ears were used as controls. This study has been approved by the Aichi Medical University Ethics Committee (18-H004).

### 2.2. Preparation of Sections from FFPE Tissue Blocks

Formalin-fixed, paraffin-embedded (FFPE) tissues were fixed with 10% neutral buffered formalin at room temperature and then set on glass slides with each section having a thickness of 3 *μ*m.

### 2.3. RNA-*In Situ* Hybridization (RNA-ISH)

*MMP2* mRNA expression level in tissue specimens from patients with cholesteatoma was assessed by *RNA*-*in situ* hybridization. RNAscope® was used for RNA-ISH. The procedure was performed according to the manufacturer's protocol. Briefly, RNAscope 2.5 HD Reagents Kit-BROWN assay kit and the MMP2 probe cocktail (Advanced Cell Diagnostic Inc, Newark, CA) were used to detect *MMP2* mRNA by RNA-ISH. Samples were deparaffinized and then activated by boiling, followed by protease digestion using a hybEZ™ oven. Hybridization was performed by incubating slides with the positive control, negative control, and MMP2 reagent in the oven; then, signal amplification was conducted. Samples were encapsulated with xylene after H&E staining and diaminobenzidine (DAB) coloring. *MMP2* mRNA expression level was quantified as the average number of DAB signal puncta per cell using Vectra 3 (PerkinElmer Co, Waltham Massachusetts). The number of *MMP2* mRNA signal puncta was counted in randomly selected foci of 0.25 mm^2^ (0.5 × 0.5 mm) under ×400 magnification. The signal puncta per cell number was determined by dividing the total spot count by the number of cells counted. Signal puncta and cell number were automatically counted using Vectra 3 (PerkinElmer Co, Waltham Massachusetts). *MMP2* mRNA expression levels were compared among the classification groups of cholesteatoma.

### 2.4. Statistical Analysis

All statistical analyses or processing was performed using SPSS V25.0 (SPSS Inc, Chicago, IL). The Mann–Whitney *U* test was used for data involving continuous values, and the Fisher's exact test was used for categorical data. All tests were performed with a two-sided alpha level of 0.05. This study was conducted in an exploratory fashion; hence, adjustments for multiple comparisons were not performed.

## 3. Results

The clinical features of the patients are summarized in Table 1. The study cohort included 38 congenital cholesteatoma and 22 acquired cholesteatoma cases. There were 39 male and 21 female patients (male : female ratio = approximately 2 : 1), and the age range was 2 to 15 years (median: 6). Patient age was significantly higher in acquired cholesteatoma cases compared to congenital cholesteatoma (*p* < 0.001). There were no significant differences in age between the two types of congenital cholesteatoma (closed type and open type, *p* = 0.678) or between the two types of acquired cholesteatoma (pars flaccida and pars tensa, *p* = 0.230). Among congenital cholesteatoma, 15 cases were classified as stage I, 22 cases were classified as stage II, and one was classified as stage III, according to JOS staging. Among acquired cholesteatoma, nine cases were classified as stage I, 12 cases were classified as stage II, and one was classified as stage III.

**Figure 2:**
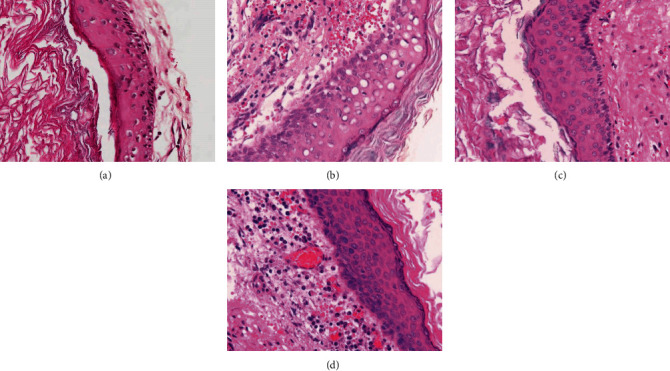
Representative histology of pediatric cholesteatoma. (a) Congenital cholesteatoma, the closed type showing a cystic structure that is lined by squamous epithelium and thin fibrous stroma with a scarce amount of inflammatory cells. (b) Congenital cholesteatoma, the open type showing a cystic structure that is lined by squamous epithelium and fibrous connective tissue with inflammation. (c) Acquired cholesteatoma, the pars flaccida type showing a cystic structure, squamous epithelium, and stroma with fibrosis. (d) The pars tensa type showing a cystic structure, squamous epithelium, and thick stroma with plasma cell-predominant inflammatory cells.

Histologically, the closed type was characterized by cystic structures surrounded by fibrous stroma and lined by squamous epithelium with a scarce number of inflammatory cells (Figure 2(a)). The open type was characterized by ruptured cysts or fragments composed of squamous epithelium and fibrous connective tissue with inflammation (Figure 2(b)). Both types of acquired cholesteatomas were characterized by pearly material consisting of keratin, squamous epithelium, and fibrous stroma with inflammatory cells (Figures 2(c) and 2(d)).

**Figure 3:**
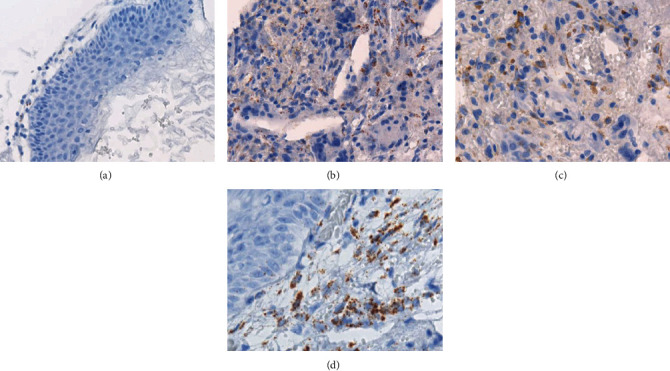
RNA-*in situ* hybridization assay in pediatric cholesteatoma, probing for *MMP2*. (a) A minute number of MMP2-positive signals were observed in congenital cholesteatoma (closed type). (b–d) MMP2-positive signals were observed in inflammatory cells in the stroma of congenital cholesteatoma (open type), pars flaccida type, and pars tensa type of acquired cholesteatoma. The MMP2 signal is shown as brown puncta.

**Figure 4:**
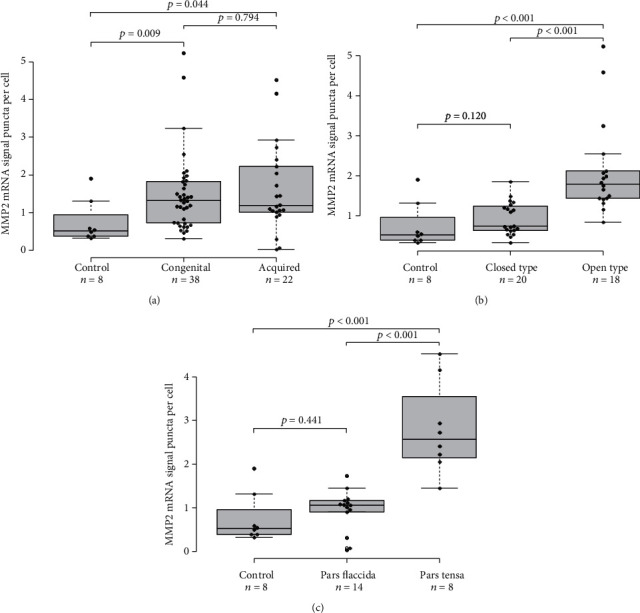
Box-and-whisker plot showing *MMP2* mRNA expression levels in cholesteatomas. The *MMP2* mRNA expression level was quantified as the average number of DAB signal puncta per cell using Vectra 3 (PerkinElmer Co, Waltham, MA). The signals and cells within the shooting range were automatically counted. The average numbers between subtypes of cholesteatoma and normal skin were compared using the Mann–Whitney *U* test, and *p* values are presented as a box-and-whisker plot. The means with standard error of the means (SEM) are shown for each group. (a) A comparison of *MMP2* mRNA expression levels in congenital cholesteatoma, acquired cholesteatoma, and normal skin from the accessory ear (shown with control). There was no significant difference in the mRNA expression level of *MMP2* between congenital cholesteatoma and acquired cholesteatoma. Congenital cholesteatoma and acquired cholesteatoma had significantly higher *MMP2* mRNA expression levels than the normal skin (*p* = 0.009 and *p* = 0.044, respectively). (b) A comparison of *MMP2* mRNA expression levels between closed and open types of congenital cholesteatoma. The open type had higher *MMP2* mRNA expression levels than the closed type (*p* < 0.001) and normal skin (*p* < 0.001). No significant difference was observed between the closed type and normal skin. (c) A comparison of *MMP2* mRNA expression levels between the pars flaccida and pars tensa types of acquired cholesteatoma. The pars tensa type had significantly higher *MMP2* mRNA expression levels than the pars flaccida type (*p* < 0.001) and normal skin (*p* < 0.001). No significant difference was observed between the pars flaccida type and normal skin.

We used RNA-ISH to identify *MMP2* mRNA expression level in the tissue specimens from patients with cholesteatoma. RNA-ISH revealed that *MMP2* mRNA signals were frequently observed in the areas with confluent inflammatory cells in open type and pars tensa type (Figures 3(b) and 3(d)). In contrast, *MMP2* mRNA signals were scarce in closed type and pars flaccida type (Figures 3(a) and 3(c)). There were no significant differences in *MMP2* mRNA expression level between congenital cholesteatoma and acquired cholesteatoma (Figure 4(a)). In congenital cholesteatoma, higher *MMP2* mRNA signals were observed in the open type than in the closed type (*p* < 0.001) (Figure 4(b)). In acquired cholesteatoma, higher *MMP2* mRNA signals were observed in the pars tensa type than in the pars flaccida type (*p* < 0.001) (Figure 4(c)).

**Figure 5:**
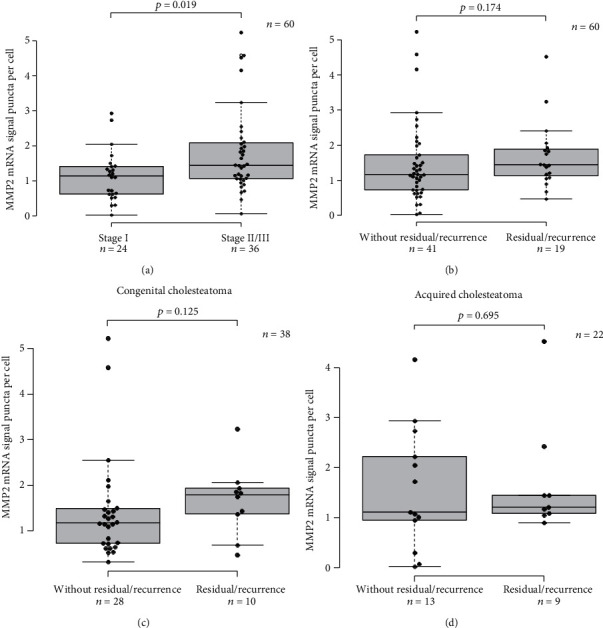
Box-and-whisker plot showing the association of *MMP2* mRNA expression levels with clinical features. (a) A comparison of *MMP2* mRNA expression levels between cholesteatomas from stage I and stage II or III. Significantly higher expression levels were observed in stage II or stage III group (*p* = 0.019). (b) A comparison of *MMP2* mRNA expression levels between cholesteatomas in this study with and without residual or recurrent lesions (*p* = 0.174). (c) A comparison of *MMP2* mRNA expression levels between congenital cholesteatoma with and without residual or recurrent lesions (*p* = 0.125). (d) A comparison of *MMP2* mRNA expression levels between congenital cholesteatoma with and without residual or recurrent lesions. No apparent trend was observed between the two groups (*p* = 0.695).

We then studied the association between the stages of cholesteatoma and tissue *MMP2* mRNA expression level. Our results revealed that higher *MMP2* mRNA signals were observed in stage II or III cholesteatoma, compared to stage I cholesteatoma (Figure 5(a)). We also studied the association between residual rate and *MMP2* mRNA expression; however, there was no significant difference in *MMP2* mRNA expression level in patients with and without residual or recurrent lesion (Figure 5(b)). Next, we analyzed the residual rate of the patients after the primary surgery according to the subtype of cholesteatoma. Relatively frequent residual lesion was observed in open type than in closed type of congenital cholesteatoma, wherein the residual rate of closed type and open type was 20% (4/20 cases) and 33% (6/18 cases), respectively (Table 2). As for the acquired cholesteatoma, there was no apparent difference in residual rate between the two subtypes; the residual rate of pars flaccida and pars tensa type was 43% (6/14 cases) and 38% (3/8 cases), respectively (Table 2). In congenital cholesteatoma, relatively higher MMP2 mRNA signals were observed in cases with residual lesion in the subsequent postoperative course, although no significant difference was observed (Figure 5(c); *p* = 0.125). In acquired cholesteatoma, there was no significant association between MMP2 mRNA signals and the presence of residual lesions (Figure 5(d); *p* = 0.695).

## 4. Discussion

Cholesteatoma is a hyperproliferative disorder of keratinocytes, characterized by a pearly keratinous material and squamous epithelium in the middle ear. As cholesteatoma progresses, it causes inflammation, ossicle-chain erosion, and temporal bone destruction. Cholesteatoma in childhood is classified into congenital and acquired cholesteatoma. Each subtype of cholesteatoma is currently considered to be derived from different origins. Acquired cholesteatoma is thought to be caused by the retraction of the eardrum, while congenital cholesteatoma is hypothesized to have resulted from the lack of involution of the epidermis [1–4]. Cholesteatoma is a clinically heterogeneous disease, with some patients showing spontaneous regression [12], while others experience an aggressive, lethal disease [13]. However, it is unclear what determines the prognosis of cholesteatoma. There is growing evidence that host inflammatory responses contribute to cholesteatoma progression. Upregulation of several inflammatory cytokines has been shown to predict worse prognosis [14]. MMP2 is a core member of the MMP family, consisting of proteolytic enzymes that degrade the extracellular matrix, and is known to play an important role in cholesteatoma progression via bone destruction and tumor cell invasion [9]. Notably, serum levels of MMP2 were reported to be associated with the degree of ossicle injury and invasion of cholesteatoma [7, 9, 10]. However, serum levels of MMP2 might be affected by systemic inflammatory conditions [15], which prompted us to quantify the amount of MMP2 in the cholesteatoma tissue. Herein, we observed higher *MMP2* mRNA expression level in the open type than in the closed type and in the pars tensa than in the pars flaccida type, which suggests that *MMP2* mRNA expression level in the cholesteatoma tissue is strongly associated with specific subtypes of cholesteatoma.

It has been previously reported that the open type has higher incidence of otitis media with effusion, involvement of tympanic membrane, and residual lesions after surgery than in the closed type [16]. Similarly, the pars tensa type has higher incidence of stapes and/or mastoid involvement and residual lesions after surgery than in the pars flaccida type [17, 18]. These studies indicated that the open type and pars tensa type exhibit an aggressive clinical behavior, and combined with our findings, this suggests that upregulation of *MMP2* mRNA level is associated with aggressive features of cholesteatoma.

*MMP2* mRNA expression level is induced by several factors, including extracellular signals such as growth factors, cytokines, mitogens, and environmental stress [19]. Keratinocytes in cholesteatoma are known to secrete inflammatory cytokines, inducing fibroblasts and lymphocytes to produce growth factors and cytokines. In turn, these secreted cytokines and growth factors promote keratinocyte proliferation and invasion. This positive-feedback loop is assumed to relate to the pathogenesis and progression of cholesteatoma [20]. Considering the high expression of *MMP2* mRNA in the aggressive types of cholesteatoma, it is plausible that MMP2 plays a critical role in the progression of aggressive cholesteatoma.

Our findings were in concordance with the report by Morales et al. showing that MMP2 protein expression assessed by immunohistochemistry (IHC) was higher in invasive cholesteatomas with clinical complications [7]. However, they also noted that MMP2 protein expression was observed in the epithelial cells, which contradicts our observations that *MMP2* mRNA signals were almost exclusively found in fibroblasts and inflammatory cells in the stroma. Although we cannot explain this discrepancy, such differences between protein and mRNA expression level of *MMP2* were reported by a previous study [21] and might be caused by increased protein turnover or nonspecific antibody binding. Benefiting from the unique “double-Z” probes, we believe that the RNAscope method is more sensitive and objective than IHC [22].

As mentioned above, it is widely believed that congenital and acquired cholesteatomas have different mechanisms. However, Olszewska et al. hypothesized that congenital and acquired cholesteatomas are derived from the same origin, and some congenital cholesteatomas progress into acquired cholesteatoma, based on the identical cytokeratin expression pattern between congenital and acquired cholesteatomas [23]. Our findings support this theory, as we demonstrated that open type of congenital cholesteatoma has shared pathology with pars tensa type of acquired cholesteatoma, in that they both have upregulated *MMP2* mRNA expression level.

The clinical significance of *MMP2* mRNA expression level has yet to be determined. Although *MMP2* mRNA expression level tends to be upregulated in congenital cholesteatoma patients who turned out to have residual disease after primary surgery, significant difference was not observed (*p* = 0.125). Our sample size might be too small to draw any conclusion about the association between *MMP2* mRNA expression and clinical outcomes. Furthermore, the clinical outcome of cholesteatoma is affected by the selection of surgical approach and technical difficulty of eradication and does not necessarily reflect biological characteristics of each case [17]. Still, we believe that our data indicates that MMP-2 expression is correlated with the biology of cholesteatoma, and it is possible that MMP2 expression might be used as a prognostic marker or surrogate marker for recurrence of cholesteatoma. Further studies are needed to elucidate this issue.

Upregulation of *MMP2* mRNA level in aggressive cholesteatoma raises the possibility that MMP2 may be a promising therapeutic target. Lehman et al. evaluated the effectiveness of ilomostat, an MMP2 inhibitor, as a therapeutic agent in an animal model of cholesteatoma [24]. The authors observed a trend toward less severe atelectasis in ilomostat-treated ears; however, MMP2 activity was not significantly diminished by ilomostat administration and hypothesized that crust accumulation may have impeded drug delivery to the lesion. Further studies are needed to address the effectiveness of MMP2 inhibition in alleviating cholesteatoma.

There are several limitations to this study. Firstly, although several past studies on cholesteatoma explored not only MMP2 but also IL-6, other MMPs, TREM-2, and EGFR, this study focused only on the expression of *MMP2* mRNA. The abovementioned factors were shown to be associated with MMP2 expression level [11, 25, 26]. The underlying mechanisms of MMP2 expression in cholesteatoma can be clarified by analyzing the expression levels of these genes. Secondly, the study population was too small to draw a conclusion. Lastly, the storage period of the FFPE samples ranged from 2 to 10 years, which might have affected the RNA quality of the specimens.

In conclusion, our study revealed that *MMP2* mRNA expression level is strongly associated with different subtypes of cholesteatoma. *MMP2* mRNA expression might be used as a prognostic or surrogate marker for recurrence of cholesteatoma. *MMP2* mRNA expression level is suggested to contribute to the aggressiveness of cholesteatoma, and thus, MMP2 inhibition may be a therapeutic option for cases featuring MMP2 upregulation.

## Figures and Tables

**(a):** 

	Closed type (*n* = 20)	Open type (*n* = 18)	Pars flaccida (*n* = 14)	Pars tensa (*n* = 8)	*p* value
Gender (male/female)	11 (55.0%)/9 (45.0%)	13 (72.2%)/5 (27.8%)	9 (64.2%)/5 (35.8%)	6 (75.0%)/2 (25.0%)	0.682
Age	5.3 ± 2.36	5.4 ± 1.46	11 ± 3.77	8.8 ± 3.65	<0.001
Lesions (right/left)	10 (50.0%)/10 (50.0%)	7 (38.8%)/11 (61.2%)	9 (64.2%)/5 (35.8%)	2 (25.0%)/6 (75.0%)	0.302

**(b):** 

	Congenital cholesteatoma (*n* = 38)		Acquired cholesteatoma (*n* = 22)		*p* value
Age	5.3 ± 1.96		9.9 ± 3.74		<0.001
	Closed type (*n* = 20)	Open type (*n* = 18)			
	5.3 ± 2.36	5.4 ± 1.46			0.678
			Pars flaccida (*n* = 14)	Pars tensa (*n* = 8)	
			11 ± 3.77	8.8 ± 3.65	0.230

**Table 2:** Residual rate of the patients after the primary surgery.

	Closed type (*n* = 20)	Open type (*n* = 18)	Pars flaccida (*n* = 14)	Pars tensa (*n* = 8)
Residual or recurrence	4	6	6	3
Without residual or recurrence	16	12	8	5
Residual rate	20.0%	33.3%	42.9%	37.5%

## Data Availability

The data used to support the findings of this study are available from the corresponding author upon request.
